# Supplementary material for: Effectiveness of and Inequalities in COVID-19 Epidemic Control Strategies in Hungary: A Nationwide Cross-Sectional Study
Source: Healthcare (Basel). 2023 Apr 25;11(9):1220. doi: 10.3390/healthcare11091220 (PMC10178097; doi:10.3390/healthcare11091220)
Supplement: Supplementary file 1 [file healthcare-11-01220-s001.zip › healthcare-2332497-supplementary.pdf]

Table S1. Post-hoc analysis for the chi-square test on the association between age and test confirmed SARS-CoV2 infection

age		not infected	infected	total
middle	Observed	508	82	590
	Expected	518.503	71.497	590
	Adjusted Residual	-2.097	2.097	
	P-value	0.036	0.036	
older	Observed	125	5	130
	Expected	114.246	15.754	130
	Adjusted Residual	3.103	-3.103	
	P-value	0.002	0.002	
young	Observed	230	32	262
	Expected	230.251	31.749	262
	Adjusted Residual	-0.055	0.055	
	P-value	0.956	0.956	
total	Observed	863	119	982
	Expected	863	119	982

Table S2. Post-hoc analysis for the chi-square test on the association between age and testing for SARS-CoV2 infection

age		not tested	tested	total
middle	Observed	368	238	606
	Expected	401.595	204.405	606
	Adjusted Residual	-4.571	4.571	
	P-value	<0.001	<0.001	
older	Observed	115	17	132
	Expected	87.476	44.524	132
	Adjusted Residual	5.435	-5.435	
	P-value	<0.001	<0.001	
young	Observed	185	85	270
	Expected	178.929	91.071	270
	Adjusted Residual	0.913	-0.913	
	P-value	0.361	0.361	
total	Observed	668	340	1008
	Expected	668	340	1008

Table S3. Post-hoc analysis for the chi-square test on the association between age and contact tracing

age		not contact traced	contact traced	total
middle	Observed	23	67	90
	Expected	29.318	60.682	90
	Adjusted Residual	-2.519	2.519	
	P-value	0.012	0.012	
older	Observed	3	4	7
	Expected	2.28	4.72	7

	Adjusted Residual	0.596	-0.596	
	P-value	0.551	0.551	
young	Observed	17	18	35
	Expected	11.402	23.598	35
	Adjusted Residual	2.355	-2.355	
	P-value	0.018	0.018	
total	Observed	43	89	132
	Expected	43	89	132

Table S4. Post-hoc analysis for the chi-square test on the association between age and vaccination against COVID-19

age		not vaccinated	vaccinated	total
middle	Observed	338	268	606
	Expected	317.429	288.571	606
	Adjusted Residual	2.65	-2.65	
	P-value	0.008	0.008	
older	Observed	113	19	132
	Expected	69.143	62.857	132
	Adjusted Residual	8.199	-8.199	
	P-value	<0.001	<0.001	
young	Observed	77	193	270
	Expected	141.429	128.571	270
	Adjusted Residual	-9.175	9.175	
	P-value	<0.001	<0.001	
total	Observed	528	480	1008
	Expected	528	480	1008

Table S5. Post-hoc analysis for the chi-square test on the association between level of infection and test confirmed SARS-CoV2 infection

education		not infected	infected	total
primary	Observed	152	9	161
	Expected	141.49	19.51	161
	Adjusted Residual	2.776	-2.776	
	P-value	0.006	0.006	
secondary	Observed	585	90	675
	Expected	593.203	81.797	675
	Adjusted Residual	-1.73	1.73	
	P-value	0.084	0.084	
tertiary	Observed	126	20	146
	Expected	128.308	17.692	146
	Adjusted Residual	-0.634	0.634	
	P-value	0.526	0.526	
total	Observed	863	119	982
	Expected	863	119	982

Table S6. Post-hoc analysis for the chi-square test on the association between level of education and testing for SARS-CoV2 infection

education		not tested	tested	total
primary	Observed	139	26	165
	Expected	109.345	55.655	165
	Adjusted Residual	5.34	-5.34	
	P-value	<0.001	<0.001	
secondary	Observed	453	241	694
	Expected	459.913	234.087	694
	Adjusted Residual	-0.994	0.994	
	P-value	0.320	0.320	
tertiary	Observed	76	73	149
	Expected	98.742	50.258	149
	Adjusted Residual	-4.269	4.269	
	P-value	<0.001	<0.001	
total	Observed	668	340	1008
	Expected	668	340	1008

Table S7. Post-hoc analysis for the chi-square test on the association between level of education and contact tracing

education		not contact traced	contact traced	total
primary	Observed	6	6	12
	Expected	3.909	8.091	12
	Adjusted Residual	1.351	-1.351	
	P-value	0.177	0.177	
secondary	Observed	32	68	100
	Expected	32.576	67.424	100
	Adjusted Residual	-0.25	0.25	
	P-value	0.803	0.803	
tertiary	Observed	5	15	20
	Expected	6.515	13.485	20
	Adjusted Residual	-0.785	0.785	
	P-value	0.433	0.433	
total	Observed	43	89	132
	Expected	43	89	132

Table S8. Post-hoc analysis for the chi-square test on the association between level of education and vaccination against COVID-19

education		not vaccinated	vaccinated	total
primary	Observed	87	78	165
	Expected	86.429	78.571	165
	Adjusted Residual	0.097	-0.097	

	P-value	0.922	0.922	
secondary	Observed	337	357	694
	Expected	363.524	330.476	694
	Adjusted Residual	-3.612	3.612	
	P-value	<0.001	<0.001	
tertiary	Observed	104	45	149
	Expected	78.048	70.952	149
	Adjusted Residual	4.611	-4.611	
	P-value	<0.001	<0.0010	
total	Observed	528	480	1008
	Expected	528	480	1008
